# Unclassified hepatocellular adenoma with histological brown pigment deposition and serum PIVKA-II level elevation: a case report

**DOI:** 10.1186/s40792-020-00853-6

**Published:** 2020-05-07

**Authors:** Kouki Hoshino, Norifumi Harimoto, Ryo Muranushi, Kei Hagiwara, Takahiro Yamanaka, Norihiro Ishii, Mariko Tsukagoshi, Takamichi Igarashi, Akira Watanabe, Norio Kubo, Kenichiro Araki, Ran Tomomasa, Sumihito Nobusawa, Shinichi Aishima, Osamu Nakashima, Ken Shirabe

**Affiliations:** 1 Department of General Surgical Science, Division of Hepatobiliary and Pancreatic Surgery, Graduate School of Medicine, Gunma University, 3-39-22 Showamachi, Maebashi, Gunma 371-8511 Japan; 2 Department of Human Pathology, Graduate School of Medicine, Gunma University, Maebashi, Gunma 371-8511 Japan; 3 Department of Pathology and Microbiology, Faculty of Medicine, Saga University, Nabeshima, Saga, 849-8501 Japan; 4 Department of Clinical Laboratory Medicine, Kurume University Hospital, Kurume, Fukuoka, 830-0011 Japan

**Keywords:** Hepatocellular adenoma, PIVKA-II, Lipofuscin granule, Hepatectomy

## Abstract

**Background:**

Hepatocellular adenoma (HCA) is conventionally considered a rare benign liver tumor, but advanced studies have revealed that HCA is heterogeneous, and may include a type that is prone to malignant transformations. Differentiation between well-differentiated hepatocellular carcinoma and focal nodular hyperplasia is necessary to diagnose hepatocellular adenoma through imaging; however, the tumor marker of hepatocellular carcinoma, protein induced by vitamin K absence, or antagonist II (PIVKA-II), is rarely positive in hepatocellular adenoma.

**Case presentation:**

A 44-year-old woman presented to our hospital with complaints of loss of appetite and weight loss. Multidetector row computed tomography revealed a liver tumor (diameter, 80 mm) that was enhanced in the arterial phase. Her serum PIVKA-II level was very high (3327 mAU/mL). Based on the enlargement of the mass and the results of the diagnostic imaging, hepatocellular adenoma or hepatocellular carcinoma was suspected, and we considered the possibility of a malignant transformation due to the high level of serum PIVKA-II; thus, we performed hepatectomy. Histological examination showed brown pigment deposition in the hepatocytes, which was determined to be lipofuscin granules. Based on immunohistochemical findings, the diagnosis was unclassified hepatocellular adenoma. Immunohistochemical examinations revealed that the adenoma cells in the tumor were positive for PIVKA-II. Her serum PIVKA-II level returned to normal after the resection.

**Conclusions:**

We present a case of unclassified hepatocellular adenoma with brown pigment deposition and elevation of serum PIVKA-II level. For the differentiation of liver tumors with high levels of PIVKA-II and hypervascular mass, hepatocellular adenoma should be considered.

## Background

Hepatocellular adenoma (HCA) is a rare benign epithelial liver tumor often observed in young women taking oral contraceptives. It is associated with oral contraceptive intake, glycogen storage disease types I and III, and a history of excess androgen exposure [1, 2]. HCA was recently classified by the World Health Organization into three main groups based on correlations between their genotype and phenotypes as follows: (1) HNF1A-inactivated HCA (H-HCA) (30–35%), (2) inflammatory HCA (IHCA) (35–40%), and (3) β-catenin activated HCA (b-HCA) (10–15%) and β-catenin-activated IHCA (b-IHCA) (10–15%) [3–5]. Although the HCA classification is widely accepted, unclassified HCA accounts for 5–10% of all HCAs, which have not been characterized by a specific phenotype, radiological features, or genetic mutations. Furthermore, there have been few reports of elevated serum protein induced by lower levels of vitamin K, antagonist-II (PIVKA-II), or des-γ-carboxy prothrombin (DCP) in HCA. We herein report a case of unclassified HCA with a histological deposition of brown pigment and elevated serum levels of PIVKA-II.

## Case presentation

**Fig. 1:**
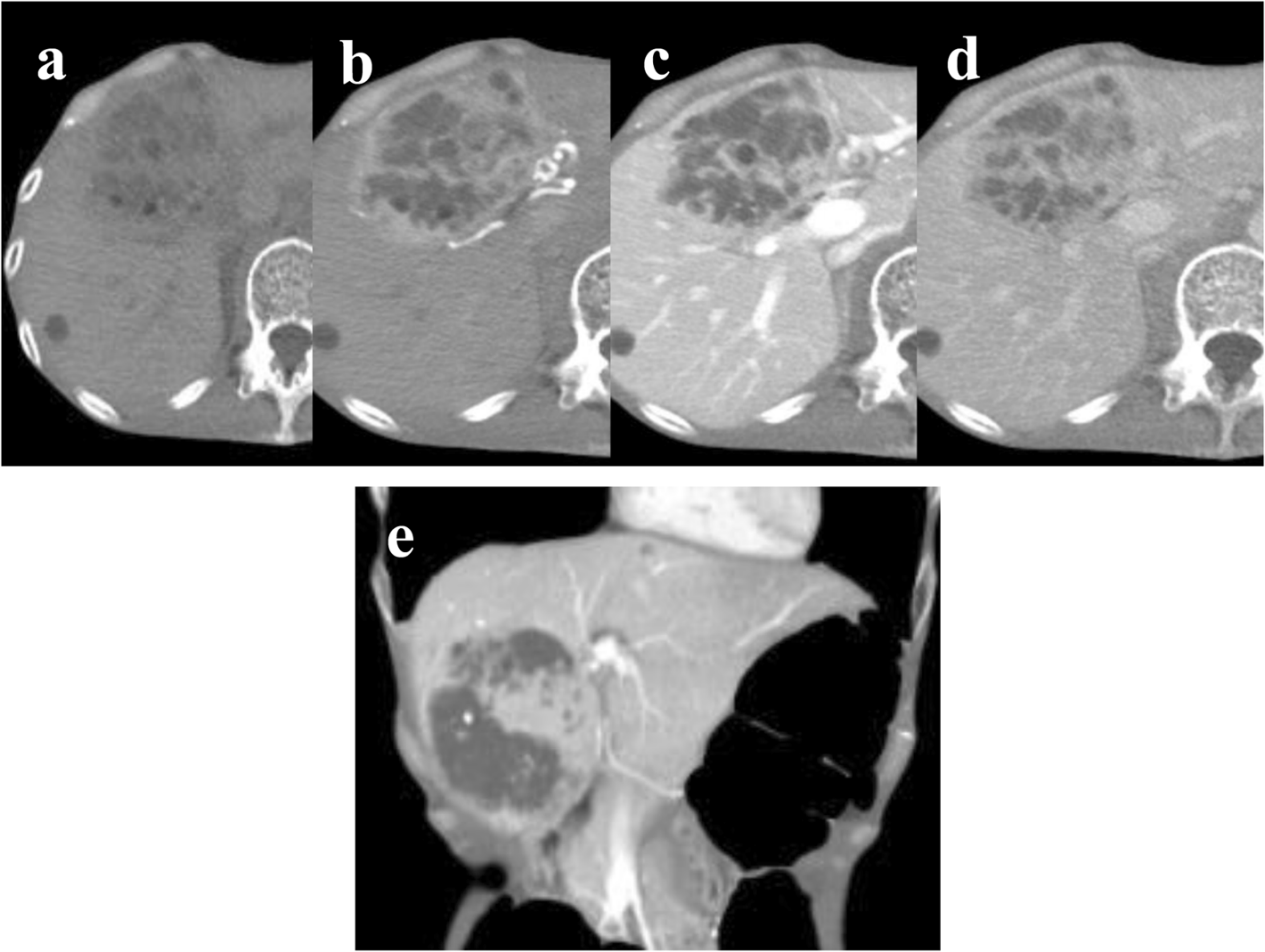
Computed tomography (CT) imaging. **a** Plain CT showed a slight low-density tumor (diameter, 80 mm) in segments S4 + S5. **b** Contrast-enhanced CT of the arterial phase showed a hypervascular mass. **c** Portal phase: the contrasting effect continued. **d** Delayed phase: the tumor looked similar to a normal liver. **e** Coronal section in the portal phase: the irregular non-contrasting area was detected inside of the tumor and accompanied by calcification, cystic parts, fatty deposition, and suspected partial hemorrhagic necrosis

**Fig. 2:**
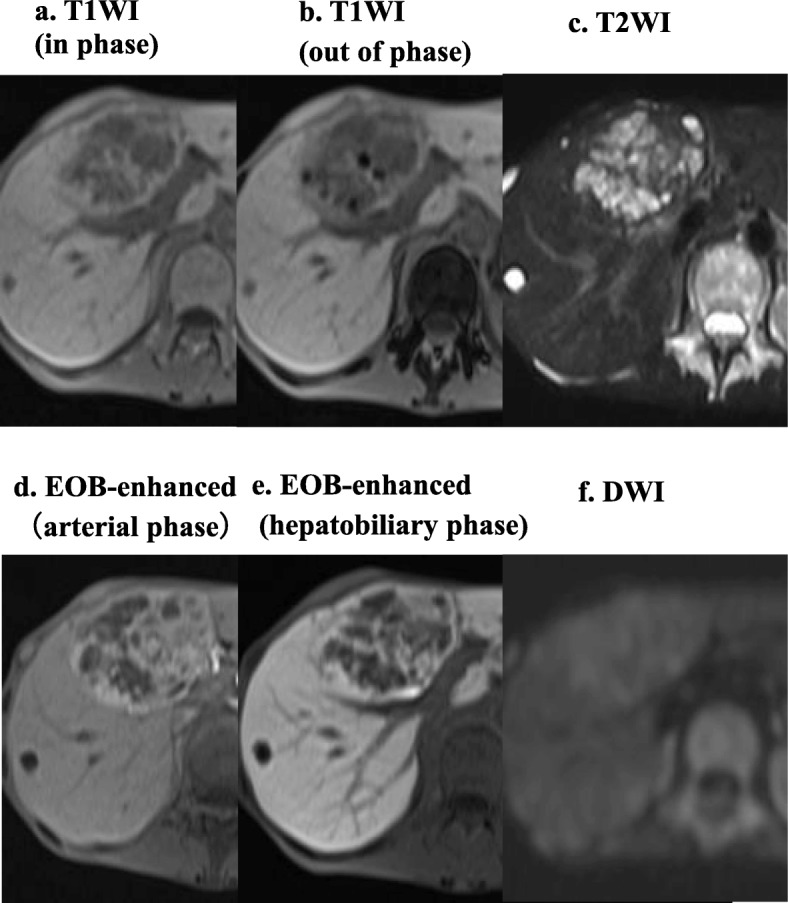
Magnetic resonance imaging. **a** T1-weighted image (in phase) showing isodense signal intensity. **b** T1-weighted image (out of phase) showing low signal intensity. The tumor inside had fatty deposition. **c** T2-weighted image. The central part of the tumor showed high density, and it was accompanied by a cystic lesion. **d** Gadolinium ethoxybenzyl diethylenetriamine pentaacetic acid (Gd-EOB-DTPA)-enhanced image (artery phase). The tumor was enhanced similar to the results of computed tomography imaging. **e** Gd-EOB-DTPA-enhanced image (hepatobiliary phase). The enhancement remained. **f** Diffusion-weighted images showed slightly high density

A 44-year-old woman exhibiting weight loss and loss of appetite was referred to our hospital because of a lesion in the liver that was detected by ultrasonography. The patient’s body mass index (BMI) was 10.9 kg/m^2^. She had no history of liver disease, pregnancy (gravida 0, para 0), oral contraceptive, or anabolic steroid use. Her laboratory findings on admission showed mild liver and kidney dysfunction (Child-Pugh class A) (Table 1), and viral markers were negative. Her serum PIVKA-II level was extremely high (3327 mAU/mL; normal value, ≤37 mAU/mL), but her serum α-fetoprotein level was within normal limits. Multidetector row computed tomography (CT) revealed a liver tumor that was 80 mm at its largest diameter and had a high density with enhancement in segments S4 + S5 (Fig. 1a–d). It was enhanced during the arterial phase and obscured during the delayed phase (Fig. 1b–d). The inside of the tumor had cystic parts, calcification, fatty deposition, and suspected partial hemorrhagic necrosis (Fig. 1e). It was isointense compared to a normal liver on T1-weighted magnetic resonance imaging (MRI) (Fig. 2a), and hyperintense in accordance with the non-enhanced area of the tumor on T2-weighted MRI (Fig. 2b). Similar to the CT imaging results, after gadolinium ethoxybenzyl diethylenetriamine pentaacetic acid administration, the tumor was enhanced heterogeneously during the arterial phase and obscured during the transitional phase (Fig. 2d). The lesion was almost isointense compared to a normal liver at the hepatobiliary phase (Fig. 2e). Diffusion-weighted images showed a slightly high intensity compared to the background liver. No central scar was detected on CT or MRI. No uptake of 2-fluoro-2-deoxy-d-glucose was identified in the mass on positron emission tomography CT (not shown).

**Table 1:** Laboratory data on admission

WBC count, /μL	4500	Cre, mg/dL	0.82
RBC count, × 10^4^/μL	384	Na, mEq/L	134
Hb, g/dL	12.2	K, mEq/L	3.6
Ht, %	37.0	Cl, mEq/L	91
PLT, × 10^4^/μL	33.0	Ca, mg/dL	8.8
PT, %	119	CRP, mg/dL	0.10
PT-INR	0.89	HbA1c, %	5.7
APTT, s	28.8	CEA, ng/mL	5.1
T-bil, mg/dL	0.6	CA19-9, IU/mL	11
AST (GOT), IU/L	36	AFP, ng/mL	6.8
ALT (GPT), IU/L	35	AFP-L3, %	< 0.5
ALP, IU/L	342	PIVKA-II, mAU/mL	3327
γ-GTP, IU/L	44	HBs-Ag	(−)
Amy, IU/L	22	HBc-Ag	(−)
TP, g/dL	6.4	HBc-Ab	(−)
Alb, g/dL	3.7	HCV-Ab	(−)
BUN, mg/dL	34		

*AFP* α-fetoprotein, *ALB* albumin, *ALP* alkaline phosphatase, *ALT* alanine aminotransferase, *APTT* activated partial thromboplastin time *AST* aspartate aminotransferase, *BUN* blood urea nitrogen, *CA19-9* carbohydrate antigen 19-9, *CEA* carcinoembryonic antigen, *Cre* creatinine, *γ-GTP* c-glutamyl transpeptidase, *Hb* hemoglobin, *Hct* hematocrit, *LDH* lactate dehydrogenase, *Plt* platelets, *PT* prothrombin time, *RBC* red blood cell, *T-bil* total bilirubin, *TP* total protein, *WBC* white blood cell

**Fig. 3:**
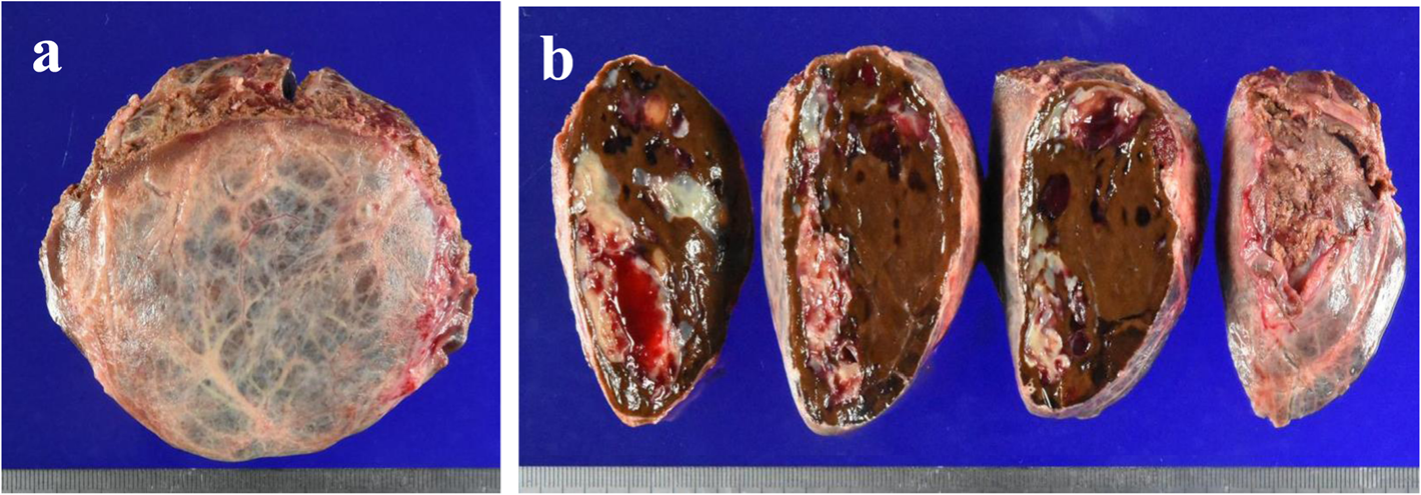
Macroscopic appearance of the resected tumor. **a** The size of the tumor was 90 × 48 mm. **b** The tumor inside showed hemorrhaging and “map-like” white and yellow parts

**Fig. 4:**
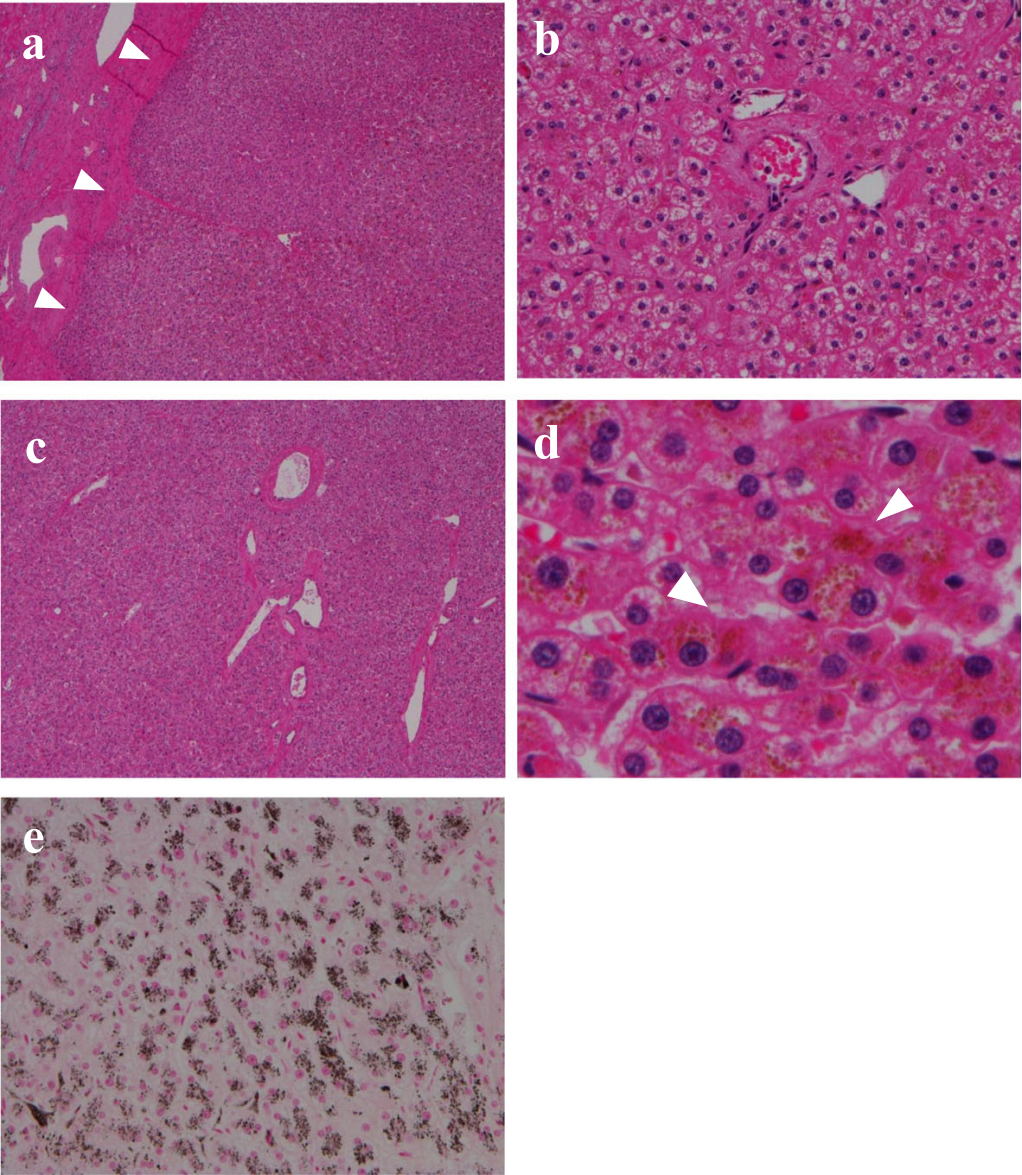
Histopathological findings. **a** The tumor consisted of two to three layers of hepatocyte (H&E stain, LPF). **b** The hepatocytes had poor atypia (H&E stain, HPF). **c** Abnormal muscular vessels developed in the tumor (H&E stain, HPF). **d** Brown pigment deposition in the hepatocytes (white arrow; H&E stain, HPF). **e** The pigment was stained black (Fontana-Masson stain). H&E hematoxylin-eosin, LPF low-power field, HPF high-power field

**Fig. 5:**
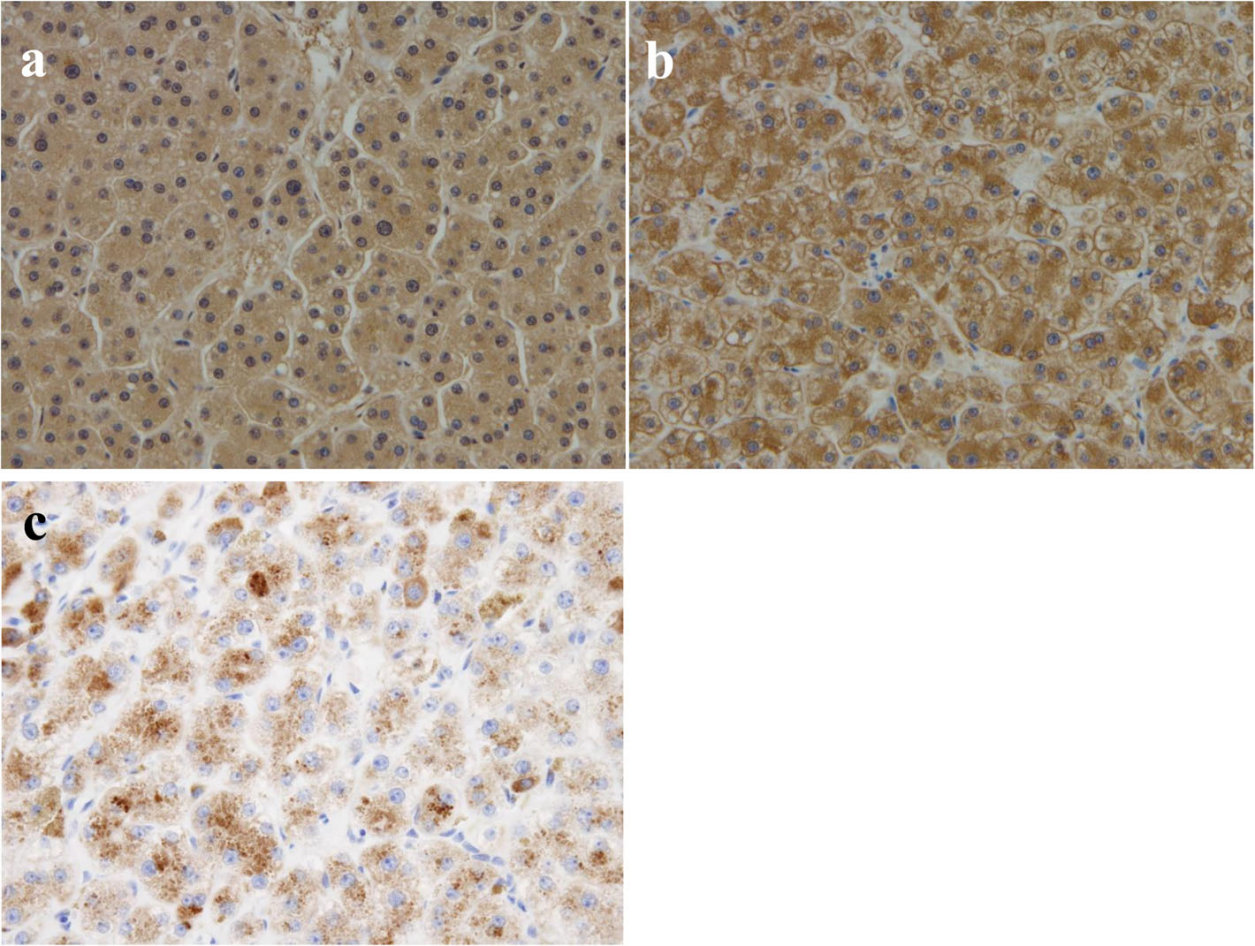
Immunohistochemical stain findings. **a** Positive LFABP expression. **b** Positive GS expression. **c** Positive PIVKA-II expression. LFABP liver fatty acid-binding protein, GS glutamine synthetase, PIVKA-II protein induced by vitamin K absence or antagonist-II

Radiologically, HCA and hepatocellular carcinoma (HCC) are considered different diseases, but accurate diagnosis based on the imaging findings alone is difficult. According to some studies [3–5], when managing HCA, a biopsy is recommended for diagnosing tumor subtype when the tumor is smaller than 5 cm. However, the risk of malignant transformation is high in patients with tumors bigger than 5 cm and in male patients; thus, hepatectomy is recommended in these groups. In this case, even though the patient was female, the tumor had already grown larger than 5 cm and hemorrhagic necrosis was suspected via CT, so we decided against a biopsy because it might have led to further bleeding. Furthermore, this patient also had a significantly elevated level of serum PIVKA-II, which indicated the possibility of malignant transformation. Therefore, we performed hepatectomy of segments S4 + S5 for the definite diagnosis and treatment. The resected specimen had a 90 × 66-mm tumor in segments S4 + S5 (Fig. 3a, b). In the high-density fields of the CT imaging results, hematoxylin-eosin staining of the pathological specimen showed 2–3 layers of hepatocytes, which had poor atypia and small nucleoli (Fig. 4a, b). In the low-density fields of the CT imaging results, “map-like” fibrosis was detected, and bleeding, hemosiderin deposition, calcification, and ossification were also partially observed. In the tumor, portal tracts and bile ducts were absent, and abnormal muscular arteries had developed (Fig. 4c). Fatty deposition and inflammatory cell infiltration were observed in the hepatocytes, but no sinusoidal dilatation was recognized. In addition, brown pigment deposition was observed in the hepatocytes (Fig. 4d). The brown pigment was stained black by Fontana-Masson stain (Fig. 4e), and showed negative results to iron staining (not shown); thus, it was believed to be lipofuscin pigment. Immunohistochemical examinations revealed positive liver fatty acid-binding protein (LFABP) expression (Fig. 5a). The expressions of C-reactive protein (CRP), serum amyloid A (SAA), and nuclear β-catenin were negative (not shown), whereas the expression of glutamine synthetase (GS) was positive (Fig. 5b). PIVKA-II (MU-3, 1:500 dilution; EIDIA, Tokyo, Japan) was positive in the adenoma cells (Fig. 5c); thus, we believe that PIVKA-II was produced by the tumor. We finally diagnosed the patient with unclassified HCA. After tumor resection, the patient’s serum PIVKA-II decreased to normal levels. Furthermore, her anorexia gradually improved, and she gained about 9 kg in 3 months. She had no recurrences for 3 months.

## Conclusions

The incidence of HCA in Western countries is 3–4 per 100,000 [3, 4]. In contrast, HCA is a very rare disease in Asia, particularly in Japan. The risks of hemorrhage (20–25%) and malignant transformation (4% in women and 47% in men) have been well reported [2, 6–10]. HCA presents as a hypervascular mass; thus, diagnostic differentiation from HCC is sometimes difficult [11].

Characteristic with immunohistochemical staining, the positive expression of LFABP in normal hepatocytes becomes negative in HNF1α-inactivated HCA. HCC rarely accompanies this type of tumor. Furthermore, this type often occurs in multiple tumors; thus, it is called liver adenomatosis. In the inflammatory HCA, SAA and CRP are the inflammatory proteins that are overexpressed, and it is characterized histologically by inflammatory cell infiltration, bile ductular proliferation, and sinusoidal dilatation. This type usually occurs in overweight women and is often accompanied by bleeding. The β-catenin-activated types (b-HCA and b-IHCA) often show cellular and structural atypia due to the activation of the Wnt/β-catenin signaling pathway. Immunohistochemical staining indicated nuclear overexpression of β-catenin and GS, which is downstream from the β-catenin pathway. This type of mutation is caused by a deletion of CTNNB1, and most hotspot mutations in exon 3 lead to high levels of β-catenin activation and GS overexpression. However, exon 3 S45 and exon 7/8 mutations lead to moderate and weak activation of this pathway, respectively, resulting in diffuse heterogenous GS staining and weak patchy GS staining [5]. Patients with these mutations in exon 3 are at an increased risk of a malignant transformation compared to those with other subtypes. The unclassified type does not show a clear gene mutation or histologic characteristics, and cases are difficult to diagnose based on heavy bleeding and necrosis alone. In our case, there were no clear histological characteristics; hence, the diagnosis was the unclassified type. However, the unclassified type can include an unknown subtype. In our case, the nuclear expression of β-catenin was negative, but the GS expression was positive; thus, we believe that this tumor had similar properties to those of the β-catenin-activated type. Furthermore, in one reported case, the HCA was diagnosed at the time of the excision, but there was a recurrence; therefore, the diagnosis was changed to well-differentiated HCC [12]. Hence, careful follow-up was necessary in this case.

A discriminative point for this case is the very high level of deposition of brown pigment in the adenoma cells. We used Fontana-Masson and iron stains, and the brown pigment was believed to be lipofuscin granules. Lipofuscin consists of lipid-containing residues from lysosomal digestion arising from aging and damage; hence, lipofuscin is called the “waste pigment” or “age pigment” [13–16]. Furthermore, it has been reported to have an indirect connection with carcinogenesis [15, 16]. In our case, we believe that her nutritional disorder (BMI 10 kg/m^2^) may be related to the lipofuscin granule deposition. However, cases of brown pigment deposition in adenoma cells have been reported as pigmented HCA in Eastern countries and Japan [17–20], and it is associated with the HNF-1α-inactivated and β-catenin-activated types, which signify malignant transformation [17, 18]. Although our case was the unclassified type, pigmented HCA was possible; thus, we need to consider the risks of malignant transformation.

In this case, the serum level of PIVKA-II was very high, but it is usually negative in cases of HCA. A PubMed search of the keywords “hepatocellular adenoma” and “PIVKA-II or DCP” (i.e., HCA cases showing high serum levels of PIVKA-II) yielded seven reports (Table 2) [19, 21–25]. The average age of the patients was 34 years (range, 21–57), with a male to female ratio of 1:1 when we include the present case. This represents an atypical number of men when compared to previous reports, where 85% of cases occur in young women. The average BMI was 20.35 kg/m^2^, and there was comparative slimness. The mean PIVKA-II was 3254.3 mAU/mL (range, 66–10,100 mAU/mL). There have been two reported cases of malignant transformation of HCA [18, 21]. The concept of subtype classification was proposed in 2010, and there were only four cases with reference to the subtype, and two cases of lipofuscin deposition [24]. For treatment, hepatectomy was performed in all cases because a malignant transformation was possible, as indicated by the high level of serum PIVKA-II. Because of the low number of cases, we were unable to determine a clear association between high serum PIVKA-II level and malignant transformation, deposition of lipofuscin granules, and subtype classification. In HCC, the production PIVKA-II was believed to be caused by vitamin K insufficiency, vitamin K metabolic disorders, selective defects in the γ-carboxylase enzyme (which prevents the production of normal prothrombin), and/or cytoskeletal changes that impair vitamin K uptake as the hepatocytes undergo malignant transformation [26]. However, in this case, because the serum PIVKA-II level decreased to a normal level after resection and immunohistochemical examination revealed a positive PIVKA-II result, the tumor was believed to be a source of PIVKA-II, which was thought to occur in patients with vitamin K metabolic disorders and dysfunctional γ-carboxylation in adenoma cells such as HCC. Thus, PIVKA-II may be a useful index of recurrence in this case.

**Table 2:** Reported cases of hepatocellular adenoma with high levels of serum PIVKA-II

No	Authors (year)	Age (years)	Sex	BMI	Tumor sites in the liver	Maximum tumor size (cm)	PIVKA-II (mAU/mL)	HCA classification	Malignant transformation	Lipofuscin deposition
1	Uto et al. [21]	21	M	23.9	Single, posterior segment	10 × 8	2200	No data	(−)	(−)
2	Ito et al. [22]	57	F	–	Single, lateral segment	10 × 10 × 8	3502	No data	(+)	(−)
3	Seyama et al. ^23^	27	F	–	Multiple, S5, S8, S7	12 × 10 (S5)	6647	No data	(−)	(−)
4	Sakamoto et al. [23]	21	M	–	Multiple, S2, S3, S4, S5/7, S7	3.6	107	No data	(−)	(−)
5	Iguchi et al. [19]	46	F	19.8	Multiple, lateral segment, S4, S1	10 × 8.5 (lateral)	10,100	β-catenin (+), but exon 3 (−)	(+)	(−)
6	Koya et al. [24]	34	M	26.8	Single, posterior segment	7 × 10	76.0	Concurrent Inflammatory+β-catenin activated	(−)	(+)
7	Koya et al. [24]	24	M	Normal	Single, lateral segment	7 × 5.5	75.4	Inflammatory	(−)	(−)
8	Our case (2019)	44	F	10.9	Single, segments S4 + S5	9 × 4.8	3327	Unclassified	(−)	(+)

*BMI* body mass index, *PIVKA-II* protein induced by vitamin K absence or antagonist II, *HCA* hepatocellular adenoma

In conclusion, we presented a case of unclassified HCA with brown pigment deposition and serum PIVKA-II level elevation. To differentiate liver tumors with high PIVKA-II levels and hypervascular masses by imaging, HCA should be considered.

## Data Availability

Data sharing is not applicable to this article as no datasets were generated or analyzed during the current study.

## Abbreviations

SAA: Serum amyloid A
BMI: Body mass index
CRP: C-reactive protein
CT: Computed tomography
DCP: Des-γ-carboxy prothrombin
GS: Glutamine synthetase
HCA: Hepatocellular adenoma
HCC: Hepatocellular carcinoma
HNF1α: Hepatocyte nuclear factor 1α
LFABP: Liver fatty acid-binding protein
MRI: Magnetic resonance imaging
PIVKA-II: Protein induced by vitamin K absence or antagonist-II
